# Protective Effects of Oral Astaxanthin Nanopowder against Ultraviolet-Induced Photokeratitis in Mice

**DOI:** 10.1155/2017/1956104

**Published:** 2017-09-28

**Authors:** Fumiya Harada, Tetsuro Morikawa, Anton Lennikov, Anthony Mukwaya, Mira Schaupper, Osamu Uehara, Rie Takai, Koki Yoshida, Jun Sato, Yukihiro Horie, Hiroyuki Sakaguchi, Ching-Zong Wu, Yoshihiro Abiko, Neil Lagali, Nobuyoshi Kitaichi

**Affiliations:** ^1^Division of Oral Medicine and Pathology, Department of Human Biology and Pathophysiology, School of Dentistry, Health Sciences University of Hokkaido, Tobetsu, Japan; ^2^School of Dentistry, College of Oral Medicine, Taipei Medical University, Taipei, Taiwan; ^3^Department of Ophthalmology, Institute for Clinical and Experimental Medicine, Linkoping University, Linkoping, Sweden; ^4^Laboratory of Biomedical Cell Technologies, School of Biomedicine, Far Eastern Federal University, Vladivostok, Russia; ^5^Division of Disease Control and Molecular Epidemiology, Department of Oral Growth and Development, School of Dentistry, Health Sciences University of Hokkaido, Tobetsu, Japan; ^6^The Research Institute of Personalized Health Sciences, Health Sciences University of Hokkaido, Tobetsu, Japan; ^7^Department of Ophthalmology, Faculty of Medicine and Graduate School of Medicine, Hokkaido University, Sapporo, Japan; ^8^Health Care Laboratory, FUJIFILM Corporation, Tokyo, Japan; ^9^Department of Dentistry, Taipei Medical University Hospital, Taipei, Taiwan; ^10^Department of Dentistry, Lotung Poh-Ai Hospital, Yilan, Taiwan; ^11^Department of Ophthalmology, Health Sciences University of Hokkaido Hospital, Sapporo, Japan

## Abstract

**Purpose:**

Astaxanthin (AST) has a strong antioxidant cellular membrane chaperone protective effect. Recently, a water-soluble nanosized AST (nano-AST) form was produced, which is expected to improve the efficacy of oral intake effects. The purpose of this study was to examine whether oral nano-AST has therapeutic effects on UV-induced photokeratitis in mice.

**Methods:**

C57BL/6 mice were administered twice with either nano-AST, AST oil, lutein, or bilberry extracts 3 hours before and shortly before UV irradiation (dose: 400 mJ/cm^2^). The corneas were collected 24 hours after irradiation and stained with H&E and TUNEL. NF-*κ*B, dihydroethidium (DHE), COX-2, p-I*κ*B-*α*, TNF*α*, and CD45 expression were evaluated through immunohistochemistry, Western blot analysis, and qPCR.

**Results:**

Corneal epithelium was significantly thicker in mice orally administered with nano-AST than in the others (*p* < 0.01), with significantly less NF-*κ*B nucleus translocation (*p* < 0.001), and significantly fewer TUNEL cells (*p* < 0.01). Weaker DHE signals were detected in the nano-AST group (*p* < 0.05) relative to the others. Furthermore, reduced inflammation and decreased cell death in corneal tissue were observed in the nano-AST group, as indicated by a reduction in the expression of COX-2, p-I*κ*B-*α*, TNF*α*, and CD45.

**Conclusions:**

Oral administration of nano-AST demonstrated a protective effect on UV-induced photokeratitis via antioxidative, anti-inflammatory, and antiapoptotic activity.

## 1. Introduction

Exposure of the eye to ultraviolet B (UVB) radiation can lead to photokeratitis, a condition which is associated with upregulated expression of inflammatory mediators such as nuclear factor- (NF-) *κ*B and prostaglandin E2 (PGE2/COX2) as part of the prostaglandin-endoperoxide synthase (PTGS) system [1]. Acute UVB exposure affects all layers of the cornea and especially the epithelium [2] through inducing apoptosis and necrosis in corneal cells [3]. Previous reports indicated that UVB irradiation at 400 mJ/cm^2^ to mouse corneas is a useful model for studying acute photokeratitis and for testing the potency of antioxidant compounds [3].

Astaxanthin (AST; 3,30-dihydroxy-b,b-carotene-4,40-dione), a carotenoid without vitamin A activity [4, 5], has potential clinical applications due to its antioxidant activity, which is higher than that of *β*-carotene and *α*-tocopherol [4, 6]. Moreover, its pharmacological effects are reported, including antitumor, anticancer, antidiabetic, and anti-inflammatory activities [6–9]. Furthermore, a previous study suggests that oral AST might ameliorate metabolic syndrome in obese mice [8]. Its cell protective effects were demonstrated in the liver and vocal cords as well [10, 11]. AST exhibits reactive oxygen species (ROS) scavenging activity [12] and inhibits UVB-induced apoptosis in keratinocytes [13]. In the eye, AST attenuates retinal damage by reducing apoptosis of retinal ganglion cells in mice through inhibiting oxidative stress [14] and light-induced retinal damage [15]. Furthermore, AST decreases retinal oxidative stress in streptozotocin-induced diabetes [16], reduces retinal ischemia damage [17], and inhibits cell death of retinal ganglion cells under various stresses [18] in murine models. In humans, AST oral supplementation is reported to increase superoxide scavenging activity of aqueous humor [19].

Humans have consumed food products that are natural sources of AST, such as salmon, crabs, and seaweed, since ancient times without any known side effect or toxicity. In 1999, pure AST was approved as a dietary supplement by Food and Drug Administration (FDA) in the United States [20]. AST is partially absorbed by the intestinal mucosal cells. However, the lipophilicity of AST causes limited bioavailability of AST due to incomplete first-pass metabolism and reaching systemic circulation [21].

Recently, however, AST has been successfully produced as nanoemulsion droplets. Meor Mohd Affandi et al. exposed water/AST oil solution to high-speed centrifugation at high pressure (800 bars) to produce stabile AST oil nanodroplets (nano-AST) with a diameter of 150–160 nm. The chemical composition of AST is not altered; the nanodroplets do not aggregate and can be further dissolved in water [22].

Reduced self-oxidation and prolonged shelf life of the nano-AST compound are reported, and potential higher bioavailability is suggested. [22] FUJIFILM (Tokyo, Japan) confirmed increased serum concentration of nano-AST and its prolonged half-life in rats upon oral administration compared to AST dissolved in oil [23].

We previously reported that AST exhibits a dose-dependent anti-inflammatory effect [24, 25] and inhibits the production of inflammatory mediators of NF-*κ*B downstream pathway, by reducing NF-*κ*B activation and tumor necrosis factor-a (TNF*α*) production *in vitro* [26].

In this study, we set out to determine whether oral nano-AST has potential therapeutic effects on UV-induced photokeratitis in mice and to evaluate the protective effect comparable to commonly used antioxidants, including lutein, water-soluble bilberry extract, and AST dissolved in oil (AST oil).

## 2. Materials and Methods

### 2.1. Care of Animals

For the present study, 8–10-week-old C57BL/6J male mice were obtained from Sankyo Labo Service Corporation Inc. (Sapporo, Japan). Mice were maintained under specific pathogen-free conditions in a licensed animal care facility at the Health Sciences University of Hokkaido (Sapporo, Japan). Experiments were approved by the animal experiment committee of the Health Sciences University of Hokkaido. All procedures involving animals were performed according to the Regulations for the Care and Use of Laboratory Animals at the Health Sciences University of Hokkaido and by the ARVO resolution on the use of animals in research.

### 2.2. Treatments and UVB Irradiation

The following substances were used:

1. nano-AST (ASP-1; Lot: F4X03, FUJIFILM Corporation, Tokyo, Japan; 0.5, 5, and 50 mg/kg, double-distilled water (DDW));
2. AST oil (ASTOTS-10O; Lot: 150121-100; Takeda Shiki, Kashiwa, Japan; oil);
3. Marigold extract (lutein; Flora GLO; Lot: UE014040117; DSM Nutrition Japan, Tokyo, Japan; oil);
4. bilberry extract (anthocyanidin; dried bilberry extract, ET; Lot: 31584/M1; DDW).

The ratio and dosages of AST oil, lutein, and bilberry extract of AST: lutein: bilberry = 1 : 1 : 10 were extrapolated based on reports used as food supplementation in the human eyes; AST oil: 6 mg/day, lutein: 6–10 mg/day, and bilberry extract: 120 mg/day [21, 27–29].

Initially, to determine the effective concentration of nano-AST, UVB-exposed animals were administrated either with nano-AST (0.5, 5, and 50 mg/kg) or DDW (positive control). Nonirradiated and nontreated animals served as negative control (naïve). Afterwards, nano-AST protective effect (50 mg/kg) on murine UV-induced photokeratitis was compared to AST oil (50 mg/kg), lutein (50 mg/kg), and bilberry extract (500 mg/kg) as well as naïve control group. Drugs/compound/treatment was orally administrated using soft mouse feeding needles 3 hours before and immediately prior UV irradiation. Mice were anesthetized intraperitoneal (i.p.) with pentobarbital (50 mg/kg; Sigma-Aldrich, St. Louis, MO, USA) and UVB irradiated (290–320 nm) at a dose of 400 mJ/cm^2^ using FS-20 Fluorescent lamp (Panasonic, Osaka, Japan). At the experimental endpoint (24 hours after treatment), animals were scarified (pentobarbital, 100 mg/kg, i.p.) and tissue samples were harvested.

### 2.3. Histology and Immunohistochemistry

The corneas were harvested, fixed with 10% formaldehyde overnight at 4°C, and embedded into paraffin. Sagittal sections of 5 *μ*m thick were stained with hematoxylin-eosin (H&E) for morphological analysis and imaged with OLYMPUS BX50 (Olympus, Tokyo, Japan) using FLOVEL Filing System camera (Flovel, Tokyo, Japan). The epithelial thickness of the central cornea was measured by a masked observer and averaged.

Cell death was investigated through terminal deoxynucleotidyl transferase dUTP nick end labeling (TUNEL) staining using Cell Death Detection Kit (Roche Diagnostics Japan, Tokyo, Japan) according to the manufacturer's protocol. TUNEL-stained sections were imaged with Eclipse TE 2000-E (Nikon, Tokyo, Japan) using the EZ-C1 3.80 software. TUNEL-positive cells were counted and averaged.

For immunohistochemistry, deparaffinization, rehydration, and antigen retrieval by boiling sections in sodium citrate buffer (10 mM sodium citrate, 0.05% Tween 20, pH 6.0) and blocking (1% BSA, 1 hour, at room temperature (RT)) were performed. Sections were then incubated with primary antibodies, including rabbit polyclonal anti-CD45 antibody (ab10558; 1 : 100; Abcam, Cambridge, UK), rabbit polyclonal anti-COX-2 antibody (aa584-598; 1 : 100; Cayman Chemical, Ann Arbor, MI, USA), mouse monoclonal p-I*κ*B-*α* (B-9) (sc-8404; 1 : 200; Santa Cruz Biotechnology, Santa Cruz, CA, USA), and rabbit monoclonal cleaved caspase 3 (c-caspase 3); (D175; 1 : 100; Cell Signaling Technology, Danvers, MA, USA).

Stained sections were visualized (DyLight 488 or DyLight 594 secondary antibody (1 : 1000; Thermo Fisher Scientific, Waltham, MA, USA)), mounted (ProLong Diamond antifade reagent with DAPI (Invitrogen, Thermo Fisher Scientific, Waltham, MA, USA)), and imaged using LSM 700 (Carl Zeiss, Oberkochen, Germany). In resulting images, COX-2-positive cells (green) were counted using ImageJ, relative to the total number of DAPI-stained nuclei (blue). Images were randomized for analysis and quantified in a masked manner.

### 2.4. NF-*κ*B Nuclear Colocalization

Corneal tissues were embedded in optimal cutting temperature (OCT) compound, flash frozen in liquid nitrogen, and sectioned (10 *μ*m thickness). Thawed sections were washed (0.1 M PBS, RT), blocked (1% BSA, 1 hour, RT), stained against NF-*κ*B (rabbit monoclonal anti-NF-*κ*B (ab16502; 1 : 100; Abcam, Cambridge, UK)) overnight at 4°C, and washed (0.1 M PBS). Sections were incubated with a fluorescent dye-conjugated goat anti-rabbit antibody (1 : 100; Cell Signaling Technology Japan, Tokyo, Japan), mounted (ProLong Gold antifade reagent with DAPI; Invitrogen, Thermo Fisher Scientific, Waltham, MA, USA), and imaged. Colocalized (pink) signals in merged images were evaluated and extracted using Photoshop (Adobe Systems, San Jose, CA, USA). The numbers of resulting NF-*κ*B colocalized nuclei counted by a masked observer and averaged.

### 2.5. Detection of Reactive Oxygen Species (ROS)

Dihydroethidium (DHE, Sigma-Aldrich, St. Louis, MO, USA), an oxidative red fluorescent dye, was used for cytosolic superoxide anion (O_2_−) detection in OCT section by oxidation [30]. Briefly, sections were thawed and immediately applied with 30 *μ*M of DHE solution in PBS for 5 min, following washing with PBS and mild fixation using 1% PFA, 10 min. Stained sections were washed with PBS and mounted with ProLong Diamond antifade reagent with DAPI (Invitrogen, Thermo Fisher Scientific, Waltham, MA, USA).

Images were acquired with Eclipse TE 2000-E (Nikon, Tokyo, Japan), and areas identical in size, including the corneal epithelial layer and subjacent stroma, were evaluated for mean luminosity values and quantified with ImageJ (National Institute of Health, Bethesda, MD, USA). Counterstaining with DAPI was done for enhanced tissue visualization but was not used for quantification.

### 2.6. Western Blot Analysis

Tissue was homogenized by Qiagen TissueLyser LT (Qiagen, Hilden, Germany), and whole protein was extracted by Ready-Prep™ total protein extraction kit working solution, supplemented with Protease Halt Protease and Phosphates inhibitor cocktail (Bio-Rad, Hercules, CA, USA). Protein concentration was quantified (Qubit 3.0 Fluorometer, Thermo Fisher Scientific, Waltham, MA, USA), boiled (25 *μ*g of total protein in Laemmli Sample Buffer 1 : 3 volume ratio, 5 min, 95°C, Bio-Rad), and seperated by SDS-PAGE (Mini Protean Precast Acrylamide Gels, Bio-Rad). Samples were transferred to a polyvinylidene fluoride (PVDF) membrane by electroblotting (Trans-Blot Turbo Transfer Pack, Bio-Rad), followed by blockage (5% skimmed milk, 1 hour, RT, Bio-Rad). Subsequently, antibody incubation was performed using rabbit polyclonal anti-CD45 antibody (ab10558; 1 : 250; Abcam, Cambridge, UK), mouse monoclonal p-I*κ*B-*α* (B-9) antibody (sc-8404; 1 : 200, Santa Cruz Biotechnology, Santa Cruz, CA, USA), and rabbit polyclonal COX-2 antibody (aa 584-598; 1 : 200, Cayman Chemical, Ann Arbor, MI, USA). Followed by horseradish peroxidase-conjugated secondary goat anti-rabbit (AP307P, 2700944, 1 : 1000; Merck Millipore, Billerica, MA, USA) and anti-mouse antibodies (AP308P, 2688593; 1 : 1000; Merck Millipore, Billerica, MA, USA), even protein loading was verified by rabbit polyclonal anti-*β*-actin antibody (PA1-21167; 1 : 2000; Thermo Fisher Scientific). Signals were visualized with Chemiluminescence Clarity™ Western ECL substrate (Bio-Rad) according to the manufacturer's protocol and detected using LAS-500 Imaging System (General Electric, Fairfield, CT, USA).

### 2.7. RNA Isolation and Quantitative Real-Time PCR (qPCR)

Corneal tissue without scleral rim was disrupted (Qiagen TissueLyser LT, Qiagen, Hilden, Germany), RNA was extracted (RNeasy Mini Kit, Qiagen), quantified (NanoDrop 2000, Thermo Fisher Scientific, Waltham, MA, USA), and reverse transcript to cDNA according to the manufacturer's protocol (ReverTra Ace® qPCR RT Master Mix, Toyobo, Osaka, Japan). qPCR was performed for TNF*α* (mTNF*α* forward: GCCTCTTCTCATTCCTGCTTG; reverse: CTGATGAGAGGGAGGCCATT [31]) and GAPDH (mGAPDH forward: AGAACATCATCCCTGCATCC; reverse: CACATTGGGGGTAGGAACAC) using Kapa SYBR Fast for LightCycler 480 (Toyobo, Osaka, Japan). Three technical and five biological replicates were run per group

TNF*α* threshold cycle (C_T_) values were normalized to GAPDH values, and gene expression was calculated using the relative quantification method (2^−△△Ct^). Obtained data were adjusted as fold change relative to the naïve group.

## 3. Statistical Analysis

All values are expressed as the mean ± standard error of mean (SEM) for the respective groups. Statistical analyses were determined using the two-tailed Student *t*-test. A *p* value less than 0.05 was considered as significant. The following markings are used in the figures: nonsignificant (n.s.) (*p* > 0.05); (^∗^*p* < 0.05); (^∗∗^*p* < 0.01); (^∗∗∗^*p* < 0.001).

## 4. Results

### 4.1. Determining the Optimal Therapeutic Amount of Nano-AST and Dose-Dependent Protective Effect

**Figure 1:**
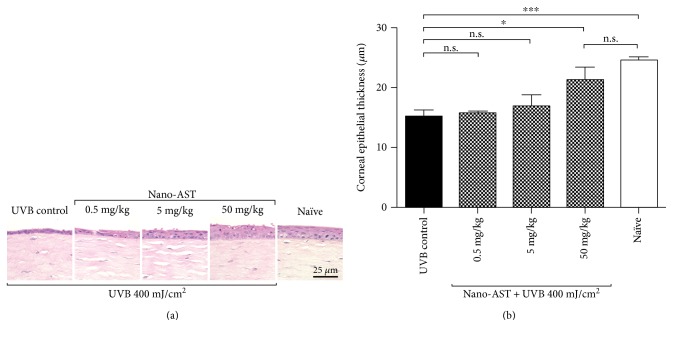
Dose-dependent effect of nano-AST on corneal epithelial thickness, 24 hours after UVB irradiation. (a) H&E staining of corneal epithelia and underlying stromal tissue. Corneal epithelium was noticeably thicker with well-preserved cellular morphology in the 50 mg/kg nano-AST group compared to the UVB control and lower concentrations (0.5 and 5 mg/kg) of nano-AST treatment. Scale bar = 25 *μ*m. (b) Quantification of corneal epithelial thickness revealed the significant protective effect of 50 mg/kg nano-AST, indicated by significantly thicker epithelium (*p* < 0.05) than untreated UVB controls. While 0.5 and 5 mg/kg nano-AST treatments did not reach significance (*p* > 0.05) when compared to the UVB control group, the overall averaged values indicate a possible dose-dependent response. *n* = 8 (eyes) per group. n.s., *p* > 0.05; ^∗^*p* < 0.05; ^∗∗∗^*p* < 0.001.

At 24 hours after UVB exposure, corneal epithelial cell layer was preserved in mice treated orally with 50 mg/kg nano-AST. In contrast, administration of 0.5 and 5 mg/kg nano-AST exhibited similar epithelial damage as well as edema in the subepithelial layer relative to the UVB control group (Figure 1(a)). Quantification of corneal epithelial thickness (Figure 1(b)) revealed that the corneal epithelium in the 50 mg/kg nano-AST group was significantly thicker compared to that of the UVB controls (*p* < 0.05), whereas epithelial thickness in the 0.5 and 5 mg/kg nano-AST-treated animals did not significantly differ from that of the UBV controls (*p* > 0.05). Based on these results, 50 mg/kg was considered as an effective concentration of nano-AST for further experiments.

### 4.2. Effect of Nano-AST Treatment Compared to AST Oil, Lutein, and Bilberry Extract

**Figure 2:**
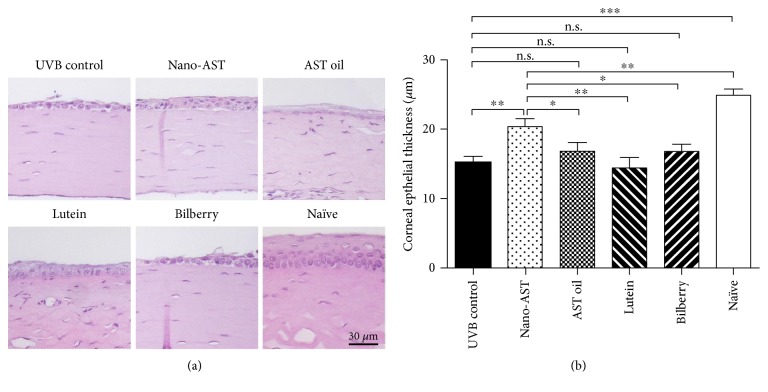
Comparison of protective effect of nano-AST, AST oil, lutein, and bilberry extract. (a) Morphological analysis of murine corneal tissue using H&E staining. Noticeable thinning and morphological and structural changes in epithelial cell layer with increased cellular infiltration of corneal stroma were observed in the UVB control, AST oil-, lutein-, and bilberry-treated groups. While thickness of corneal epithelial layer in nano-AST-treated animals was obviously thinner compared to naïve nonirradiated corneas, cellular morphology and epithelial layer structure are well preserved. Scale bar: 30 *μ*m. (b) Nano-AST (50 mg/kg) treatment resulted in significant thicker corneal epithelium compared to the UVB controls (*p* < 0.01), AST oil (*p* < 0.05), lutein (*p* < 0.01), and bilberry extract (*p* < 0.05) treatment. *n* = 8 (eyes) per group. n.s., *p* > 0.05; ^∗^*p* < 0.05; ^∗∗^*p* < 0.01; ^∗∗∗^*p* < 0.001.

Next, we investigated the protective effect of AST oil, lutein, and bilberry extract and compared it to nano-AST (Figure 2). Nano-AST treatment preserved the epithelium and resulted in milder morphological changes in the corneal surface compared to that in other groups (Figure 2(a)). No significant protective effect was observed in AST oil-, lutein-, and bilberry extract-treated groups, indicated by no detectable, significant difference (*p* > 0.05) in corneal thickness relative to the UVB control animals (Figure 2(b)). In contrast, nano-AST (50 mg/kg) significantly preserved corneal epithelial thickness compared to the UVB control group (*p* < 0.01), AST oil (*p* < 0.05), lutein (*p* < 0.01), and bilberry extract (*p* < 0.05), (Figure 2(b)).

### 4.3. Nano-AST Treatment Reduced ROS Production in Corneal Tissue

**Figure 3:**
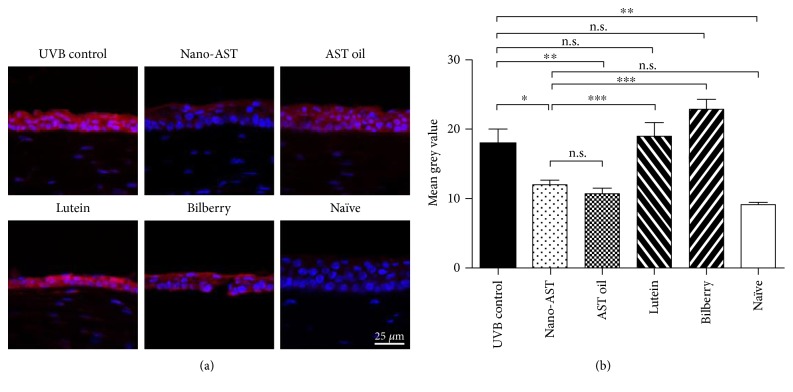
Assessment of reactive oxygen species (ROS) levels by dihydroethidium (DHE) staining. (a) Cell nuclei were stained with DAPI (blue). ROS were investigated through DHE staining. Upon reaction between ROS and DHE, DHE form, a red fluorescence, produced, namely, 2-hydroxyethidium. ROS were strongly detected in the UVB-irradiated groups, lutein, and bilberry. In contrast, the nano-AST group displayed a weak signal of ROS, with some reduction observed in AST oil as well. Scale bar = 25 *μ*m. (b) DHE staining quantification revealed significantly reduced luminosity values in nano-AST- (*p* < 0.05) and AST oil- (*p* < 0.01) treated groups. Lutein and bilberry extract administration did not result in significant reduction of ROS signal compared to the UVB control. *n* = 8 (eyes) per group. n.s., *p* > 0.05; ^∗^*p* < 0.05; ^∗∗^*p* < 0.01; ^∗∗∗^*p* < 0.001.

To investigate ROS production, harvested corneal tissue was stained with DHE (Figure 3). Immunohistochemistry revealed strong DHE expression in the UVB-irradiated groups, whereas a markedly reduced signal was detected in both nano-AST- and AST oil-treated animals (Figure 3(a)). Quantitative analysis of DHE signal (Figure 3(b)) displayed that ROS production was significantly reduced in the oral nano-AST (*p* < 0.05) and AST oil (*p* < 0.01) groups relative to the UVB-irradiated control group, while treatment with lutein or bilberry extract did not have a significant effect on ROS production in UVB-irradiated corneal tissues.

### 4.4. Nano-AST Treatment Reduced Corneal Cell Death and caspase 3-Dependent Apoptosis

**Figure 4:**
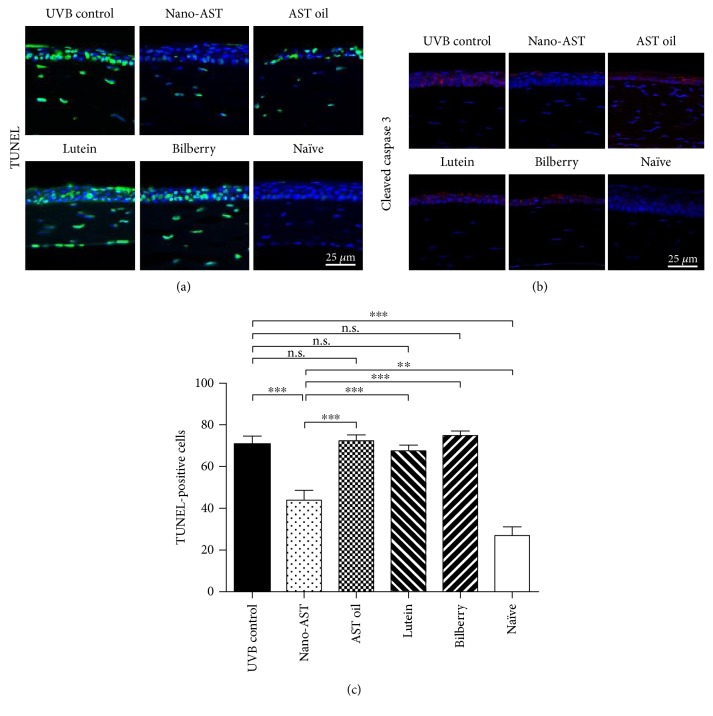
Cell death analysis in corneal tissue by TUNEL assay and cleaved caspase 3 staining. (a) Cell nuclei were stained with DAPI (blue). Cell death was evaluated by TUNEL (green) staining. High numbers of TUNEL-positive nuclei were detected in the cornea of the UVB control mice. Contrary, few TUNEL-positive nuclei were observed in nano-AST-treated mice, and AST oil administration causes a minor reduction of TUNEL-positive cells. Lutein and bilberry extract did not result in a noticeable reduction in numbers of TUNEL-positive nuclei. The nonirradiated group (naïve) showed background levels of TUNEL-positive cells, potentially associated with tissue harvesting process. (b) Cleaved caspase 3 staining findings were consistent with TUNEL staining results, with marked increased c-caspase 3-positive cells through the whole corneal epithelial layer in the UVB control, AST oil-, lutein-, and bilberry-treated groups. Nano-AST-treated animals, however, demonstrate only a few c-caspase 3-positive cell signals on the surface of the epithelial layer. No specific c-caspase 3 signals were detected in naïve corneas. (c) Numbers of TUNEL-positive cells were evaluated and averaged. Quantitative analysis confirmed the significant reduction of apoptotic cells by nano-AST (*p* < 0.001) compared to the UVB control group. AST oil, lutein, and bilberry extract had similar numbers of TUNEL-positive nuclei as the UVB control group (*p* > 0.05). *n* = 8 (eyes) per group. n.s., *p* > 0.05; ^∗∗^*p* < 0.01; ^∗∗∗^*p* < 0.001.

UVB exposure induces apoptosis in corneal cells; therefore, we evaluated the effect of nano-AST and other antioxidants on cell death. First, apoptotic cells were stained with TUNEL (Figure 4(a)). Numerous TUNEL-positive nuclei were detected in UVB-irradiated corneas, whereas only a few TUNEL-positive cells were observed in nano-AST-treated corneas. AST oil, lutein, and bilberry extract administration, however, displayed no noticeable different TUNEL staining signals compared to the UVB control. Obtained data through TUNEL staining was further supported by the apoptosis marker c-caspase 3 staining (Figure 4(b)). Quantification of the amount of TUNEL-positive cells (Figure 4(c)) revealed significant reduced number of apoptotic cell upon nano-AST administration compared to the UVB control (*p* < 0.01) and other treatment groups (*p* < 0.001). In contrast, no significant difference was detected between the UBV control group and animals treated with AST oil, lutein, or bilberry extract (*p* > 0.05, n.s.).

### 4.5. Nano-AST Treatment Reduced NF-*κ*B Activation

**Figure 5:**
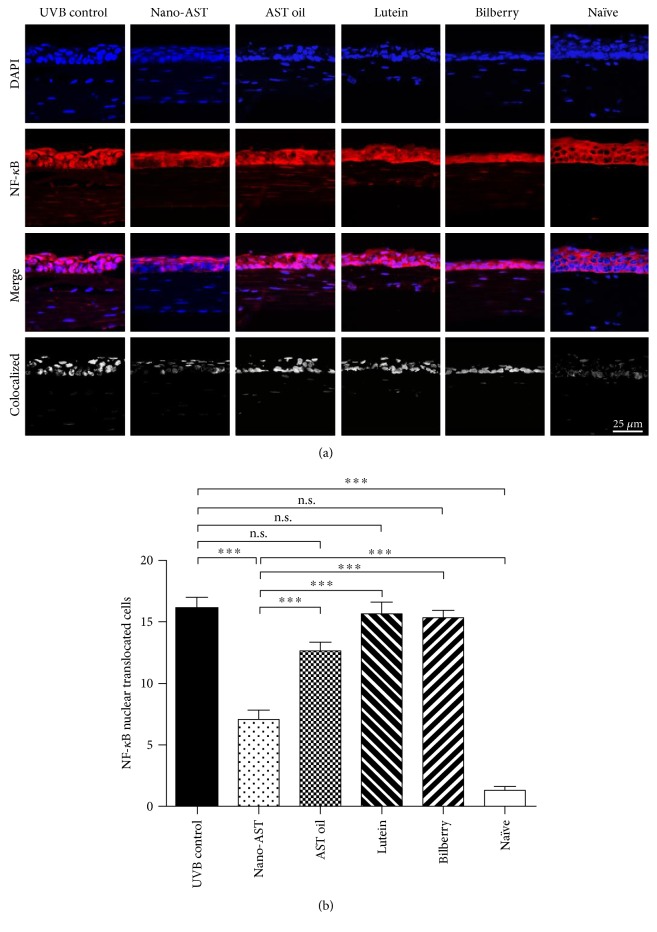
Immunofluorescent analysis of NF-*κ*B nuclear translocation. (a) UVB irradiation induces NF-*κ*B translocation into the nucleus. NF-*κ*B (red) remained in the cytoplasm in naïve mice, while nano-AST (50 mg/kg) treatment markedly reduced translocalization (pink) of NF-*κ*B in the nuclei (blue). Obtained immunohistochemistry data do not indicate significant signal change in AST oil-, lutein-, or bilberry extract-treated groups. Scale bar = 25 *μ*m. (b) For analysis, mean values of NF-*κ*B-positive cells were assessed. Numbers of NF-*κ*B-positive cells were significantly reduced in the nano-AST group relative to other irradiated groups (*p* < 0.001). *n* = 8 (eyes) per group. n.s., *p* > 0.05; ^∗∗∗^*p* < 0.001

NF-*κ*B resides in the cytoplasm in its inactive form, as observed in nonirradiated naïve mouse corneal tissues (Figure 5(a)). UVB irradiation activates the NF-*κ*B signaling pathway, resulting in NF-*κ*B translocation into the nucleus, shown in the UVB-irradiated groups (Figure 5(a)). The number of NF-*κ*B translocated into the nuclei was quantified (Figure 5(b)). Quantification revealed significantly reduced NF-*κ*B nuclear colocalization signals within the nucleus in the nano-AST group relative to the UVB control group (*p* < 0.001). Administration of AST oil, lutein, and bilberry extract did not significantly reduce NF-*κ*B translocation (*p* > 0.05) when compared to the UVB control.

### 4.6. Nano-AST Suppressed the Expression of Proinflammatory Cyclooxygenase- (COX-) 2 and Phosphorylated I*κ*B-*α* and CD45 Key Mediator in Recruitment of Inflammatory Cells

**Figure 6:**
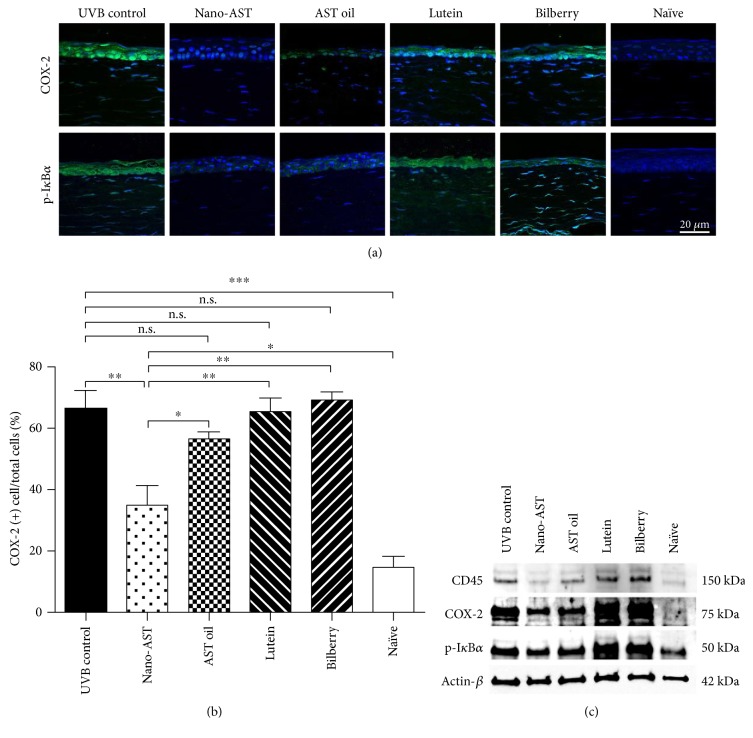
Expression level of COX-2, phospo-I*κ*B*α* (p-I*κ*B*α*), and CD45 in corneal tissue. (a) COX-2 (green) and phospho-I*κ*B*α* (green) expression in corneal tissue of treated (nano-AST, AST oil, lutein, and bilberry) and control mice (UVB control, naïve); nuclei counterstained by DAPI (blue). Scale bar = 20 *μ*m. (b) Quantitative results for COX-2 expression were calculated by a number of COX-2-positive cells per section relative to the total number of cells counted by DAPI signal and averaged. Numbers of COX-2-positive cells were significantly reduced in the nano-AST-challenged group (*p* < 0.01), but AST oil-, lutein-, and bilberry extract-treated animals revealed similar amount of COX-2-positive cells as the UVB control group (*p* > 0.05). *n* = 5 (eyes) per group. (c) CD45, COX-2, and phospho-I*κ*B-*α* expression were further confirmed by Western blot analysis. *β*-Actin was used as loading control. n.s., *p* > 0.05; ^∗^*p* < 0.05; ^∗∗^*p* < 0.01; ^∗∗∗^*p* < 0.001.

COX-2, a downstream gene of NF-*κ*B, is a crucial mediator for inflammatory cell recruitment. The expression of the proinflammatory factor COX-2 was induced upon UVB exposure (Figure 6(a), UVB control). However, clear reduction of COX-2 signal in the corneal tissue was revealed by immunohistochemistry nano-AST-treated group. While slight reduction of COX-2 signaling was observed in some AST oil-treated samples, evaluation of the percentage of COX-2-positive cells confirmed significant decrease in nano-AST- (*p* < 0.01) treated animals but revealed no significant reduction in the other treatment groups compared to the UVB control (Figure 6(b)). These results were further supported by Western blot analysis, with clear reduction of COX-2 band intensity in nano-AST-treated corneas and to lesser extent in AST oil-treated group. In contrast, lutein and bilberry extract administration did not cause decreased COX-2 expression proven by immunohistochemistry and Western blot analysis. NF-*κ*B is held in the cytoplasm by the inhibitory protein I*κ*B*α*. During NF-*κ*B activation, I*κ*B*α* is phosphorylated (pI*κ*B*α*) leading to the sequestration of NF-*κ*B, I*κ*B*α* complex and NF-*κ*B nucleus translocation. Consistent with NF-*κ*B nuclear translocation staining (Figure 5), immunohistochemistry revealed diminished pI*κ*B*α* signal in the nano-AST-treated group and decreased expression after AST oil administration compared to the UVB control (Figure 6(a)). Western blot analysis of pI*κ*B*α* expression in the cornea demonstrated slight difference between the UVB control and nano-AST band intensity (Figure 6(c)).

Furthermore, the expression of CD45 was attenuated in nano-AST and to a lesser extent in AST-treated mouse corneas (Figure 6(c)).

### 4.7. Nano-AST Treatment Reduced TNF*α* Transcription

**Figure 7:**
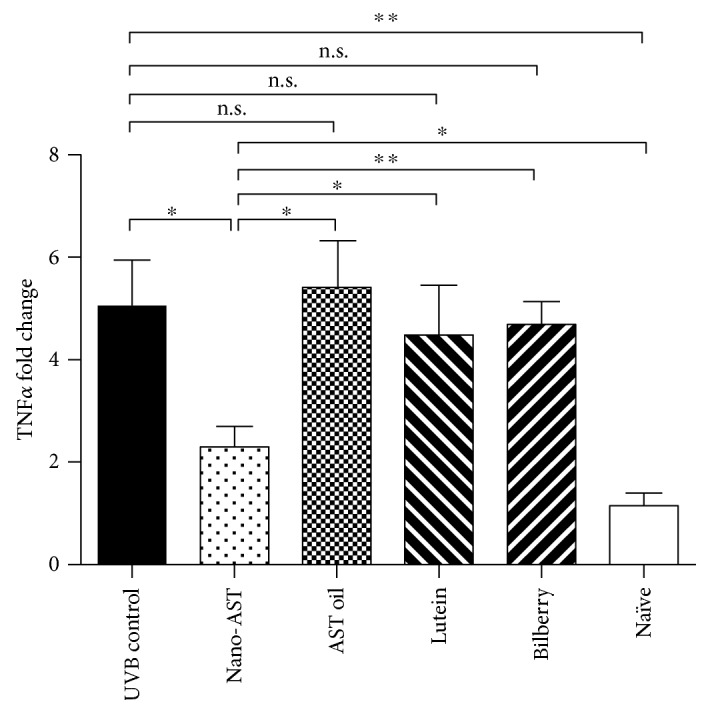
Quantitative PCR analysis of TNF*α* expression in the mouse cornea. Significant reduction in fold change TNF*α* expression when compared to the UVB control in nano-AST- (*p* < 0.05) treated group. AST oil, lutein, and bilberry extract were not significantly different from the UVB control group (*p* < 0.05). *N* = 5 (animals per group). Data represented as fold change relative to the naïve control group. n.s., *p* > 0.05; ^∗^*p* < 0.05; ^∗∗^*p* < 0.01.

The expression profile for TNF*α* in the treatment groups (UVB control, nano-AST, and AST oil) was assessed by qPCR (Figure 7()). The transcription of TNF*α* was markedly increased in the UVB control group compared to the naïve (*p* < 0.01), however, significantly reduced in the nano-AST (*p* < 0.05) group relative to the UVB control. TNF*α* gene expression was not significantly affected by AST oil, lutein, or bilberry treatment (*p* > 0.05).

## 5. Discussion

Corneal epithelium serves to protect the underlying corneal stroma, posterior eye structures, and tissues against UVB damage by absorbing a substantial amount of UV radiation. Epithelial cells have an innate antioxidant system [32] that is overwhelmed as a result of exposure to more energetic UVB light. Upon UVB exposure, ROS production transiently increases and activates cell signaling pathways [33]. Excessive UVB irradiation causes DNA and cell membrane damage that leads to the induction of necrosis and apoptosis of corneal epithelial cells as well as activation of transcription factors such as NF-*κ*B [34].

NF-*κ*B is known as one of the major transcription factors mediating inflammation and cell survival [35]. Activated NF-*κ*B induces upregulation of inflammatory mediators, enzymes, and cytokines such as COX-2, PGE2, and TNF*α* [9]. TNF*α* initiates an inflammatory positive feedback loop, resulting in NF-*κ*B activation [36]. Early tissue infiltration with inflammatory cells, primarily with CD45 and CD11b-positive leukocytes, occurs within hours after UVB exposure and causes further tissue damage [37].

AST inhibits *in vivo* activation of NF-*κ*B in endotoxin-induced uveitis (EIU) and choroidal neovascularization models [24, 25]. We previously reported that topical AST eye drops suspended in polyethylene glycol (PEG) protect against UV-induced photokeratitis through the reduction of NF-*κ*B expression and ROS activation [38]. However, lack of water solubility of AST is the limiting factor for topical use, as well as its opaque nature, which reduces vision for a short time after application. Thus, AST usage is limited to skin cosmetics products.

Furthermore, as the cornea is one of the nonvascular tissues, significantly higher blood AST level is required to achieve the desired therapeutic effect in corneal diseases after oral ingestion.

The present study indicates a protective effect of oral nano-AST administration against UV-induced acute photokeratitis. 50 mg/kg nano-AST administered orally preserved epithelial morphology and significantly reduced number of TUNEL- and NF-*κ*B-positive cells in the cornea. Immunohistochemistry, Western blot analysis, and qPCR further supported these results by indicating a significant reduction of COX-2, CD45, p-I*κ*B*α*, and TNF*α* expression, which leads to decreased inflammatory and cell death responses.

As previously reported, oral administration of nano-AST results in a 1.5–1.8 times higher plasma AST concentration compared to AST oil intake, and the plasma AST level peaked 3 hours after administration [23]. We can speculate that hydrophilic nano-AST can reach a level high enough to be effective at the ocular surface by penetration of the blood-eye barrier, and thus reaching the aqueous humor as well as the tear fluid.

To determine the relative efficacy of the nano-AST formulation, antioxidant compounds which are well known in research and commercial applications [39] were incorporated into the experimental design, such as lutein, AST oil, and bilberry extract.

Endogenously synthesized lutein is known to be detected in macular tissue of humans and some animal eyes [40]. It has been demonstrated that macular carotenoid levels can be altered through dietary manipulation and lower carotenoid levels in age-related macular degeneration (AMD) patients have been reported [41]. While the high antioxidant potency of lutein is well known and demonstrated in various cells [42] and tissues [43], its lipophilicity limits its oral bioavailability [44]. In the present study, lutein did not produce any noticeable effect on cell death, inflammation, or ROS response in the cornea.

Recent data suggested that anthocyanins are as bioavailable as other flavonoid subclasses [45], such as flavan-3-ols and flavones, which have relative bioavailabilities between 2.5% and 18.5% [46, 47]. However, anthocyanins are subjected to rapid metabolic elimination and produce many diverse breakdown products and metabolites [45], thereby limiting its usefulness for treatment of ocular diseases. In this study, both lutein and bilberry extracts were ineffective in suppressing corneal damage, in contrast to nano-AST.

AST oil reduced ROS production (comparable to nano-AST). However, AST oil did not have a significant effect on corneal epithelial cell death or inflammation. This result could be explained by the better bioavailability of nano-AST compared to AST oil [23]. Additionally, a threshold level for NF-*κ*B activation to induce an “all-or-nothing” response was reported in tissue hypoxia [48]. Therefore, partial inhibition of NF-*κ*B activation that does not reduce the activation to threshold level would have little effect on the subsequent inflammatory cascade. This contention is supported by our Western blot and histological observations, where AST oil administration slightly reduced p-I*κ*B-*α* and COX-2 expression but had minimal effect on corneal epithelial morphology, cell death markers, or TNF*α* expression profile. These results may also indicate that the effect of AST is not limited to ROS scavenging.

A recent study indicated that AST could have a direct effect on c-Jun-N-terminal kinase 1, which regulates numerous factors downstream of c-Jun, such as ATF2, SMAD4, and HSF1. These factors are highly involved in apoptosis, DNA repair, cellular proliferation, and chaperone responses, respectively [49]. Furthermore, AST has been shown to downregulate gene expression of COX-2 as well as COX-2 protein and attenuates phosphorylation of mitogen- and stress-activated protein kinase- (MSK-) 1 resulting in the decreased phosphorylation of NF-*κ*B in UVB-irradiated human keratinocytes [50]. The exact mechanism of how AST achieves such effects is not yet entirely understood. However, the reduction of endoplasmic reticulum (ER) stress or phosphorylation of MSK-1 are suggested as possible candidates [49, 50]. Further mechanistic studies of phosphorylation of c-Jun-N-terminal kinase 1 and ER stress in corneal epithelial cell cultures are required to gain a deeper understanding of the direct intracellular effects of AST that potentially becoming more prominent in nano-AST formulation.

To date, AST is not known to cause any direct toxicity even at high doses or concentrations *in vivo* [51] or *in vitro* [24]. As nano-AST is chemically identical to AST [22], it is not expected to induce direct cytotoxic effect as well. However, AST is known to accumulate in the skin, causing visible pink coloration in rats during prolonged oral consumption at doses 30 g/kg [51], while the effective concentration of nano-AST in the current study did not exceed 50 mg/kg, 600 times lower than that reported to cause a noticeable change in skin color in AST oral consumption [51]. We cannot exclude that increased solubility of nano-AST may cause a change in skin color at lower concentrations, which might be undesirable effect and limiting factor for human use. Chronical study of oral nano-AST effects on AST accumulation and color changes in mammalian skin is required to determine what amount may produce such an effect.

Our findings in nano-AST formulation not only suggest possible clinical use in situations of increased UVB exposure, such as UVB exposure risks for professional mountaineers, Arctic, and Antarctic personnel, but also suggest nano-AST potential as a supplementary and preventive treatment for wide spectrum of inflammatory and degenerative conditions in the cornea, as increased ROS production in the ocular surface associated with dry eye disease [52], keratoconus [53], Fuchs' endothelial dystrophy, and bullous keratopathy [54].

## 6. Conclusion

The present study provides evidence that nano-AST is effective in protecting the ocular surface against the detrimental effects of acute UVB exposure, with no obvious adverse side effects observed. Oral nano-AST intake might be a promising naturally derived water-soluble substance for protecting against ocular surface damage in conditions of high oxidative stress.

## Supplementary Material

Supplementary Table 1: Corneal epithelial thickness, 24 hours after UVB exposure, when treated with different doses of Nano-AST. Supplementary Table 2: Comparison of Nano-AST to other antioxidant treatments. Supplementary Table 3: Mean grey value DHE (ROS) staining in cornea. Supplementary Table 4: Quantification of TUNEL positive cells. Supplementary Table 5: Quantification of NF-?B positive nuclei. Supplementary Table 6: Quantification of COX-2 positive cells ratio. Supplementary Table 7: Quantitative PCR analysis of TNFa expression in the mouse cornea.
