# Supplementary material for: Protective Effects of Oral Astaxanthin Nanopowder against Ultraviolet-Induced Photokeratitis in Mice
Source: Oxid Med Cell Longev. 2017 Sep 28;2017:1956104. doi: 10.1155/2017/1956104 (PMC5637851; doi:10.1155/2017/1956104)
Supplement: Supplementary file 1 — Supplementary Table 1: Corneal epithelial thickness, 24 hours after UVB exposure, when treated with different doses of Nano-AST. Supplementary Table 2: Comparison of Nano-AST to other antioxidant treatments. Supplementary Table 3: Mean grey value DHE (ROS) staining in cornea. Supplementary Table 4: Quantification of TUNEL positive cells. Supplementary Table 5: Quantification of NF-?B positive nuclei. Supplementary Table 6: Quantification of COX-2 positive cells ratio. Supplementary Table 7: Quantitative PCR analysis of TNFa expression in the mouse cornea. [file 1956104.f1.pdf]

Supplementary tables

Supplementary table 1: Corneal epithelial thickness, 24 hours after UVB exposure, when treated with different doses of Nano-AST

Figure 1B	Corneal thickness (µm)	SEM	P-value
UVB control	15.2	1.09	
0.5 mg/kg	15.7	0.41	0.6533
5 mg/kg	16.9	1.94	0.4613
50 mg/kg	21.3*	2.18	0.0319
Naïve	24.5***	0.61	< 0.0001

Supplementary table 2: Comparison of Nano-AST to other antioxidant treatments

Figure 2B	Corneal thickness (µm)	SEM	P-value
UVB control	15.2	0.92	
Nano-AST	20.4**	1.1	0.0022
AST oil	16.7	1.34	0.3489
Lutein	14.3	1.58	0.6556
Bilberry	16.2	1.16	0.3214
Naive	25.0***	0.88	< 0.0001

Supplementary table 3: Mean grey value DHE (ROS) staining in cornea

Figure 3B	Mean grey value	SEM	P-value
UVB control	18.2	2.1	
Nano-AST	12.7*	0.8	0.0270
AST oil	10.9**	0.89	0.0037
Lutein	21.2	2.87	0.4081
Bilberry	23.1	1.91	0.0902
Naive	9.9**	0.91	0.0013

Supplementary table 4: Quantification of TUNEL positive cells

Figure 4C	TUNEL (+) cells	SEM	P-value
UVB control	23.9	4.1	
Nano-AST	19.1***	4.7	< 0.0001
AST oil	27.6	2.65	0.7060
Lutein	26.5	3.21	0.5179
Bilberry	29.8	2.33	0.3846
Naive	14.6***	4.19	< 0.0001

Supplementary table 5: Quantification of NF-κB positive nuclei

Figure 5B	NF-kB (+) nuclei cells	SEM	P-value
UVB control	15.2	0.9	
Nano-AST	8.1***	0.6	< 0.0001
AST oil	14.5	0.83	0.7082
Lutein	14.1	0.86	0.6231
Bilberry	14.8	0.66	0.9757
Naive	1.4***	0.3	< 0.0001

Supplementary table 6: Quantification of COX-2 positive cells ratio

Figure 6B	Cox-2 (+) cells ratio	SEM	P-value
UVB control	66.2	6.1	
Nano-AST	34.8**	6.5	0.0078
AST oil	56.4	2.4	0.1741
Lutein	65.6	4.1	0.9406
Bilberry	69.0	2.8	0.6914
Naive	14.7***	3.6	< 0.0001

Supplementary table 7: Quantitative PCR analysis of TNFα expression in the mouse cornea

Figure 7	Fold change	SEM	P-value
UVB control	2.26	0.4	
Nano-AST	1.03*	0.17	0.0242
AST oil	2.4	0.4	0.7807
Lutein	2.01	0.44	0.6875
Bilberry	2.1	0.21	0.7274
Naive	0.51**	0.11	0.0033

Values are presented as average±standard error of the mean (SEM). The P values of experimental group were compared with UVB control group. The following markings used in the manuscript figures:

non-significant; (n.s.), (p>0.05); *, (p<0.05); **, (p<0.01); ***, (p<0.001).
